# Species Diversity of Pin Nematodes (*Paratylenchus* spp.) from Potato Growing Regions of Southern Alberta, Canada

**DOI:** 10.3390/plants10020188

**Published:** 2021-01-20

**Authors:** Maria Munawar, Dmytro P. Yevtushenko, Juan E. Palomares-Rius, Pablo Castillo

**Affiliations:** 1 Department of Biological Sciences, University of Lethbridge, 4401 University Drive W, Lethbridge, AB T1K 3M4, Canada; maria.munawar@uleth.ca; 2 Institute for Sustainable Agriculture (IAS), Spanish National Research Council (CSIC), Campus de Excelencia Internacional Agroalimentario, ceiA3, Avenida Menéndez Pidal s/n, 14004 Córdoba, Spain; palomaresje@ias.csic.es (J.E.P.-R.); p.castillo@csic.es (P.C.)

**Keywords:** *Paratylenchus tateae*, *Paratylenchus neoprojectus*, plant-parasitic nematode, integrative taxonomy, morphology, DNA sequencing, phylogeny, new record, new species

## Abstract

Pin nematodes (*Paratylenchus* spp.) are polyphagous parasitic species with a wide host range and geographical distribution; their diversity is unknown in the potato growing region of Alberta, Canada. The present study aims to provide morphological and molecular characterization of three pin nematode species, namely *P. neoprojectus*, *P. tateae*, and a new species, *Paratylenchus enigmaticus* sp. nov. All of them were recovered from the potato growing region of southern Alberta. The nematodes were isolated using the sieving and flotation-centrifugation method, and their morphology was assessed by light microscopy. Molecular characterization was performed using partial 18S, D2–D3 expansion domains of the 28S and ITS ribosomal genes. This study is the first report of molecular characterization of *P. tateae* and *P. neoprojectus*, being new records from southern Alberta, and two Spanish populations of *P. tateae* comprising the first report of this species in Europe. The phylogenetic analysis of the 18S, D2–D3 expansion domains of the 28S and ITS ribosomal DNA regions underscores the importance of using molecular data for accurate species identification and clarifies the status of *P. nanus* type B and *P. sheri*. Moreover, our findings will be useful to determine the impact of pin nematodes on potato production in future field research.

## 1. Introduction

Potato is one of the most important crops in Canada, with Alberta ranking among the top provinces producing superior quality potatoes with the highest marketable yields [1]. To maintain high standards of potato production, Alberta’s farmed fields are regularly surveyed and examined for the presence of pest species. Recent reports have described the incidence of plant-parasitic nematodes (PPN) in cultivated soils of Canada [2,3,4].

*Paratylenchus* species are commonly known as pin nematodes. The short stylet species feed ecto-parasitically; however, some species feed endo-parasitically by gaining entry into lateral roots [5,6,7]. Pin nematodes are amongst the most frequently occurring PPN in Canada [8], and previous studies have reported the association of pin nematodes with forages, turf grasses, legumes, and cereal crops of Eastern and Central Canada [2,9,10,11,12,13]. Biological studies have indicated that females of *P. projectus* Jenkins [14] lay 1–2 eggs/day, with an average life cycle of 30–38 days at 20–28 °C. Additionally, several *Paratylenchus* species have a persistent survival stage (mainly the fourth stage), which helps them to maintain inoculum levels during periods of adversity [15].

*Paratylenchus* species have a wide host-range, and several short stylet species, such as *P. bukowinensis* Micoletzky [16], *P. dianthus* Jenkins and Taylor [17], *P. hamatus* Thorne and Allen [18], *P. microdorus* Andrassy [19], *P. neoamblycephalus* Geraert [20], *P. shenzhenensis* Wang, Xie, Li, Xu, Yu, and Wang [21] and *P. projectus*, cause varying degrees of damage to their hosts, including root injury and poor plant development, consequently decreasing yield and plant longevity [7,22].

Currently, the genus contains over 100 species, with only 11 reported in Canada [23,24]. *Paratylenchus* species are among the smallest PPN and this, together with their apparent similarities with other related species, makes them challenging to study and identify [25]. During a survey of potato fields, we isolated three *Paratylenchus* species. Preliminary examination revealed that all the species have advulval flaps, 4 lateral lines, and short stylets (<40 µm).

As several short stylet pin nematodes species are considered to be plant-pathogenic [22], we performed morphological/morphometrical and molecular studies on these *Paratylenchus* populations and identified them as *P. neoprojectus* Wu and Hawn [26], *P. tateae* Wu and Townshend [27], and a new *Paratylenchus* sp. that we named *P. enigmaticus* sp. nov. As the diversity of pin nematode species associated with potato growing areas of Alberta is largely unknown, the aims of the present work were to: (i) characterize the populations of *P. tateae*, *P. neoprojectus*, and *P. enigmaticus* sp. nov. found in potato growing areas of southern Alberta; (ii) update the pin nematode diversity record from Canada; (iii) study the phylogenetic relationship of these species with other pin nematodes. The results of this study will aid in distinguishing pathogenic forms from non-pathogenic species, and our findings will be useful in future field experiments to determine the impact of these PPN on potato production.

## 2. Results

### 2.1. Description of Female Paratylenchus neoprojectus Wu and Hawn

**Figure 1:**
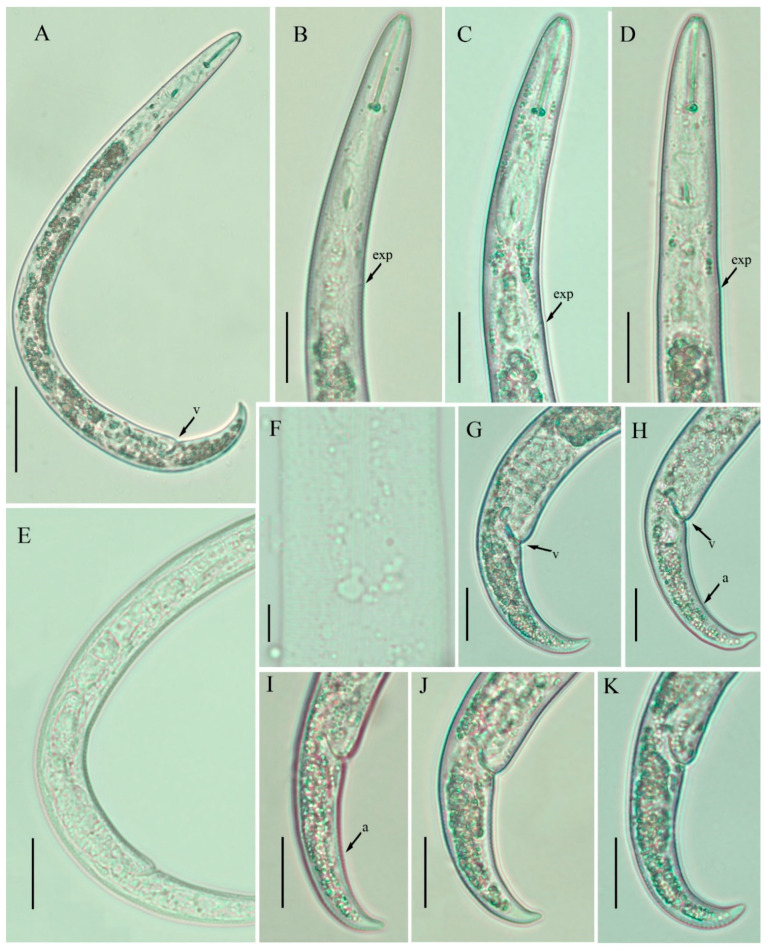
Light photomicrographs of *Paratylenchus neoprojectus* females. (**A**) Entire body; (**B**–**D**) pharyngeal regions; (**E**) posterior region with gonad; (**F**) lateral lines; (**G**–**K**) tails. Scale bars: (**A**) 50 μm; (**B**–**E**) 20 μm; (**F**) 5 μm; (**G**–**K**) 20 μm. Arrowheads: (a) Anus; (exp) excretory pore; (v) vulva.

**Figure 2:**
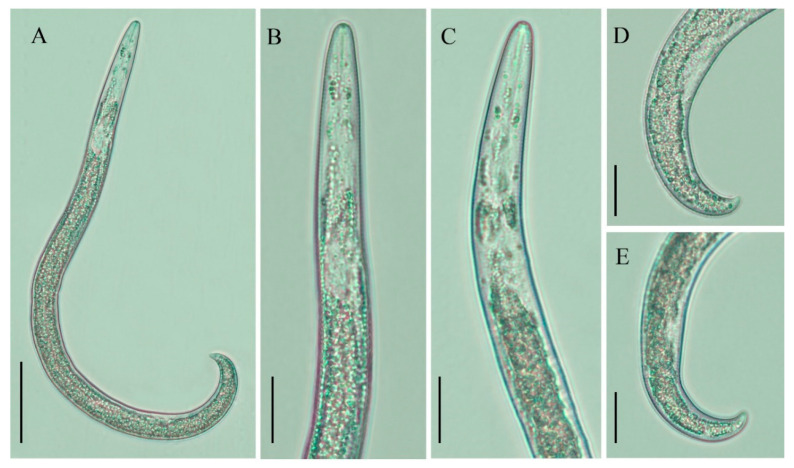
Light photomicrographs of *Paratylenchus neoprojectus* juvenile. (**A**) Entire body; (**B**,**C**) pharyngeal regions; (**D**,**E**) tails. Scale bars: (**A**) 50 μm; (**B**–**E**) 20 μm.

(Figure 1 and Figure 2; Table 1) [26].

Body slender, ventrally arcuate with a bend in the middle of the body when heat relaxed; cuticle finely annulated; lateral field equidistant with four distinct lines; lip region rounded narrow, with anterior end flattened, continuous with the rest of the body; labial framework sclerotization weak; pharyngeal region typical paratylenchoid type; stylet rigid, straight; rounded stylet knobs; dorsal pharyngeal gland opening 5.0–6.0 μm behind stylet knobs; median pharyngeal bulb large elongate, bearing distinct large valves; isthmus short slender, surrounded by nerve ring; basal bulb pyriform, pharyngeal-intestinal valve bilobed; excretory pore situated at the level or middle of pharyngeal basal bulb. Hemizonid 1–2 annuli long situated just posterior to the excretory pore. The body slightly narrower posterior to vulva; ovary outstretched, well developed, in some specimens it reaches to the level of pharynx; spermatheca and crustaformeria well developed, the columnar arrangement of crustaformeria usually not discernable; spermatheca rounded; the vulva a transverse slit occupying half of the corresponding body width; vulval lips prominent, the anterior lip protrudes further than the posterior lip; vulval flaps present, but not prominent in fresh specimens; a small, rudimentary post uterine branch present along the ventral body wall; anus indistinct; tail slender, conoid, finely annulated, and gradually tapers to form a finely rounded terminus.

#### 2.1.1. Juveniles

Only one juvenile form was detected. Individuals in this stage were similar in morphology to the adult females. However, they were characterized by the presence of weak stylet; pharynx components under-developed; genital primordium under-developed; anus indistinct; and a posterior body with a finely rounded terminus.

#### 2.1.2. Remarks

*Paratylenchus neoprojectus* was originally described from Central Alberta, Canada in the rhizosphere of alfalfa [26]. Following the formal description, the species has appeared twice in the literature [23]. The first population was reported from India [29] without morphological characterization or illustrations; only morphometrics of adult females were provided. Since overlapping morphometrical characters are common in pin nematode species [25,28], the identification of this Indian population needs to be confirmed.

The second population was reported from Iran [30], and the illustrations showed the absence of a post uterine sac (vs. present in the original description), a broadly rounded tail terminus (vs. conically or finely rounded in the original description), and a short ovary (vs. an ovary that reaches to the pharyngeal basal bulb level in the original description). All these characters are not in agreement with the original description of *P. neoprojectus*, therefore a detailed re-evaluation based on integrative taxonomy is required to determine the exact status of this population.

In 2014, Van den Berg et al. [28] reported a detailed morphological and molecular characterization of several pin nematode species from the USA and South Africa. Based on their molecular data, the authors demonstrated that *P. nanus* has two sibling species type A and type B. Comparing the morphological, molecular, and morphometrical characteristics (Figure 1 and Figure 2; Table 1), we conclude that *P. nanus* type B should be considered as *P. neoprojectus*. *Paratylenchus neoprojectus* and *P. nanus* are closely related species, but can be differentiated by the body shape (ventrally bent vs. open C-shape of *P. nanus*), position of the excretory pore (at the level or posterior to pharyngeal bulb vs. at level or anterior to pharyngeal bulb), ovary development (reaches the level of the pharyngeal basal bulb vs. short), presence of post uterine branch (vs. absent), and tail terminus morphology (conically or narrowly rounded vs. subacute to rounded, slightly indented). *Paratylenchus neoprojectus* is also close to *P. projectus* and can be differentiated from it by the lip region morphology (conical rounded vs. trapezoid), more posterior position of the excretory pore (vs. anterior), and tail terminus morphology (conically or narrowly rounded vs. often digitate terminus).

In the present study, the *P. neoprojectus* population from southern Alberta matches with the species’ original description, except for minor differences in the body length; the southern Alberta population is slightly longer than the original one (330–434 vs. 327–405 µm).

#### 2.1.3. Habitat and Locality

This population was found in the rhizosphere of *Chenopodium* sp. growing on the headland (uncultivated field margin) of a potato field, (latitude 49°48′40.5″ N; longitude—111°23′55.4″ W); Municipal District of Forty Mile County No. 8, Alberta, Canada.

### 2.2. Description of Female Paratylenchus tateae Wu and Townshend 

**Figure 3:**
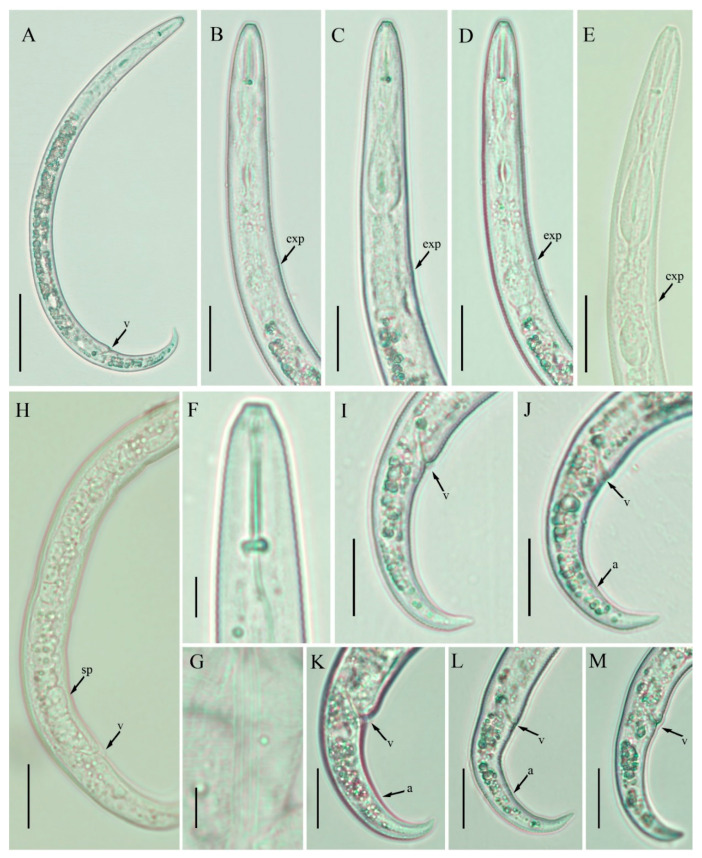
Light photomicrographs of *Paratylenchus tateae* female, Canadian population. (**A**) Entire body; (**B**–**E**) pharyngeal regions; (**F**) lip region; (**G**) lateral lines; (**H**) posterior region with gonad; (**I**–**M**) tails. Scale bars: (**A**) 50 μm; (**B**–**E**) 20 μm; (**F**,**G**) 5 μm; (**H**–**M**) 20 μm. Arrowheads: (a) Anus; (exp) excretory pore; (sp) spermatheca; (v) vulva.

**Figure 4:**
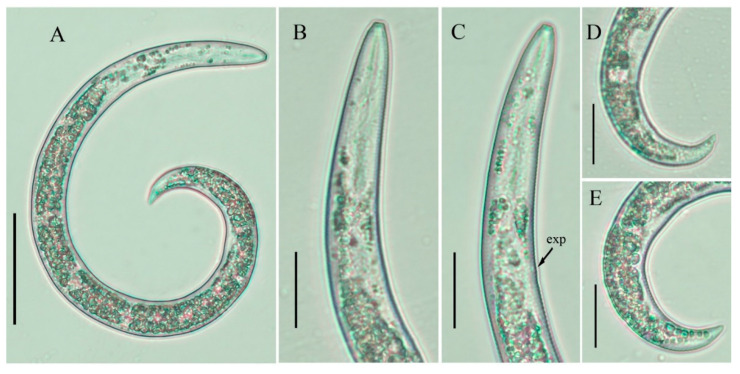
Light photomicrographs of *Paratylenchus tateae* juvenile, Canadian population. (**A**) Entire body; (**B**,**C**) pharyngeal regions; (**D**,**E**) tails. Scale bars: (**A**) 50 μm; (**B**–**E**) 20 μm.

**Figure 5:**
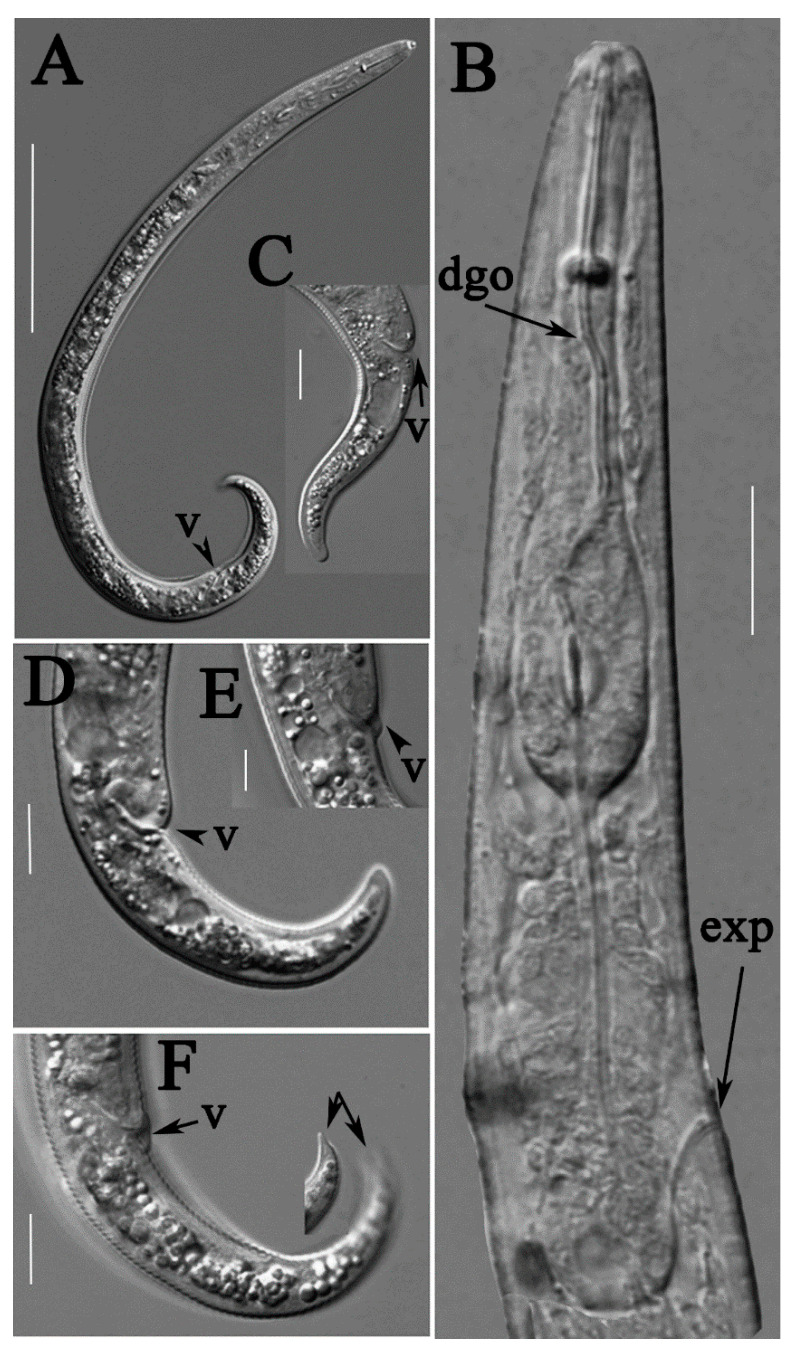
Light photomicrographs of *Paratylenchus tateae* female, Spanish population. (**A**) Entire body; (**B**) pharyngeal regions; (**C**,**D**,**F**) tails; (**E**) vulval region. Scale bars: (**A**) 50 μm; (**B**–**F**) 10 μm. Arrowheads: (dgo) Dorsal pharyngeal gland orifice; (exp) excretory pore; (v) vulva.

(Figure 3, Figure 4 and Figure 5; Table 2) [27].

Body slender, ventrally arcuate when heat relaxed; cuticle finely annulated; lateral field equidistant with four distinct lines; lip region conoid narrow, with anterior end flattened, continuous with the rest of the body; labial framework sclerotization weak; pharyngeal region, typical paratylenchoid type; stylet rigid, straight; stylet knobs, rounded; dorsal pharyngeal gland opening 4.5–6.0 μm behind stylet knobs; median pharyngeal bulb elongated, bearing distinct large valves; isthmus short slender, surrounded by nerve ring; basal bulb pyriform, pharyngeal-intestinal valve inconspicuous; excretory pore situated at the level of pharyngeal basal bulb or slightly anterior to it. Hemizonid 2–3 annuli long situated just anterior to excretory pore; body slightly narrower posterior to vulva; ovary outstretched, occasionally reflexed; spermatheca and crustaformeria not distinguishable in most of the specimens; in mature females, the spermatheca irregularly rounded without sperm; vulva a transverse slit occupying half of the corresponding body width; vulval lips prominent, the anterior lip protrudes further than the posterior lip; vulval flaps present, but not readily distinct in fresh specimens, observable in preserved specimens; a small, rudimentary post uterine branch present along the ventral body wall; anus indistinct; tail slender, conoid, finely annulated, and gradually tapers to form a finely pointed to rounded terminus, bluntly rounded terminus and tip with peg was observed in Spanish populations.

#### 2.2.1. Juveniles

Only one juvenile form was detected. This stage of individuals was similar in morphology to the adult females. However, they were characterized by the presence of weak stylet; pharynx components under-developed; genital primordium under-developed; anus indistinct; posterior body with a finely pointed terminus.

#### 2.2.2. Remarks

*Paratylenchus tateae* was originally described from Ontario, Canada, in the rhizosphere of several crops, such as corn, alfalfa, timothy, and white and red clover [27]. After the formal description, the species was reported twice in the literature [23], one of them reported in Saskatchewan [31], however Anderson and Kimpinski [32] collected samples from the same location and considered the Saskatchewan population as *P. labiosus*. The other population was described in India [29], and the author suggests that the Indian population differs from the Canadian population by smaller body length and a more posterior position of the vulva. Additionally, the description of the Indian population includes a rounded head, a disc-like lip region with prominent projecting submedian lobes, and the absence of a post uterine sac. All of these characteristics are contrary to the original description of *P. tateae*, which states the presence of a distinctive truncated lip region, weakly developed spermatheca, and a short, rudimentary post-uterine branch. Based on our current knowledge, we conclude that the Indian population presented by Bajaj [29] might not be *P. tateae*.

Morphologically and morphometrically, *P. tateae* is similar to *P. brevihastus* Wu [33]; the later species was also described in Ontario in the rhizosphere of alfalfa, blue violets, oats, red clover, and grasses. The only characters differentiating *P. tateae* from *P. brevihastus* are the absence of males and weakly developed spermatheca. We do not suggest synonymization here; we are in agreement with Van den Berg et al. [28], who stated that such actions should only be performed after careful molecular and morphological comparisons.

In the present study, we found two populations of *P. tateae* from southern Alberta, and two from Spain. All the populations match with the original description, except for minor differences in body length, as the Alberta population is slightly shorter than the original description (269–380 vs. 315–401 µm), while other characteristics are in the species variability range.

#### 2.2.3. Habitat and Locality

Two *P. tateae* populations were found in the potato growing fields of the Municipal District of Taber, Alberta, Canada. The first field was located at latitude 49°46′55.8″ N, longitude—112°21′30.8″ W, whereas the second was located at latitude 49°47′48.5″ N, longitude—112°20′49.6″ W. Two *P. tateae* populations were found in Spain, in the rhizosphere of almond and wheat, at Ariza, Zaragoza province and Alpera, Albacete province, respectively.

### 2.3. Description of Female Paratylenchus enigmaticus sp. nov.

**Figure 6:**
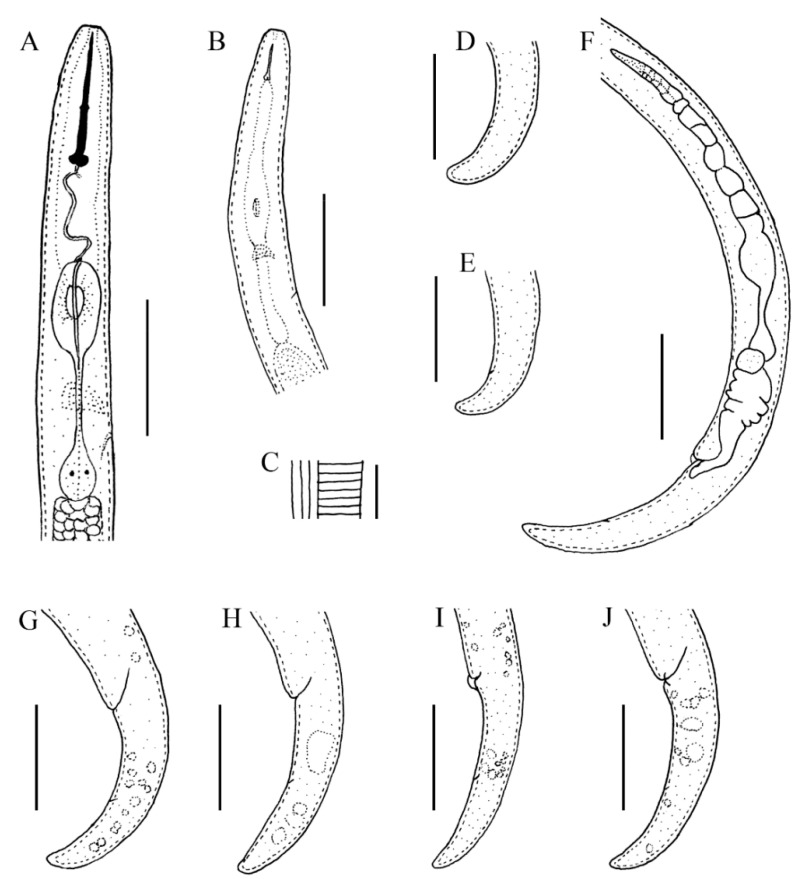
Line drawings of *Paratylenchus enigmaticus* sp. nov. (**A**) Pharyngeal region female; (**B**) pharyngeal region juvenile; (**C**) lateral field lines; (**D**,**E**) juvenile tails; (**F**) posterior region with genital branch; (**G**–**J**) female tails. Scale bars: (**A**,**B**) 20 μm; (**C**) 5 μm; (**D**–**J**) 20 μm.

**Figure 7:**
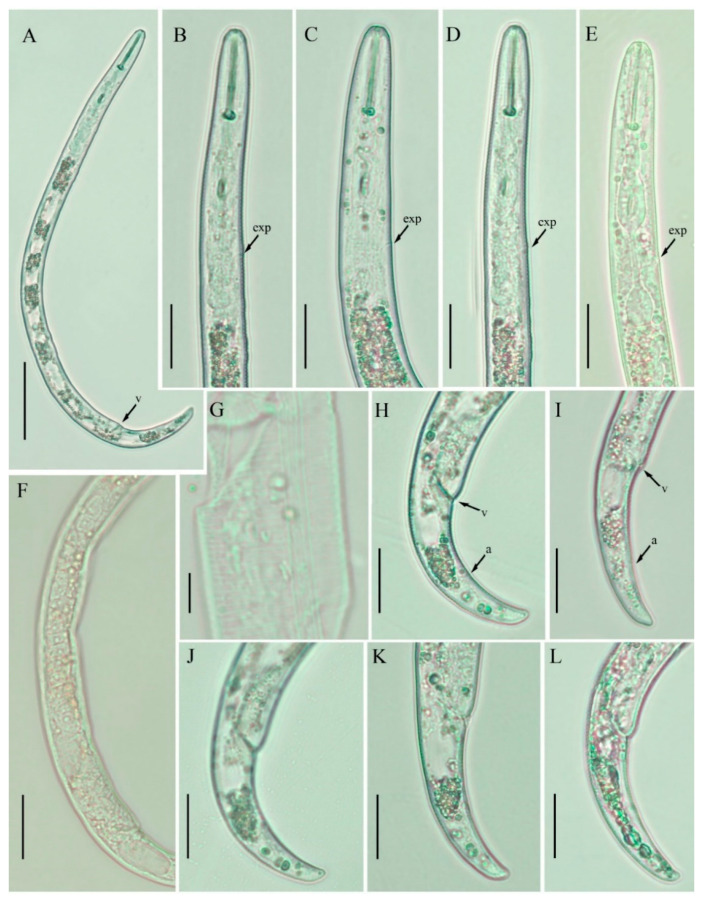
Light photomicrographs of *Paratylenchus enigmaticus* sp. nov. female. (**A**) Entire body; (**B**–**E**) pharyngeal regions; (**F**) posterior region with gonad; (**G**) lateral lines; (**H**–**L**) tails. Scale bars: (**A**) 50 μm; (**B**–**F**) 20 μm; (**G**) 5 μm; (**H**–**L**) 20 μm. Arrowheads: (a) Anus; (exp) excretory pore; (v) vulva.

**Figure 8:**
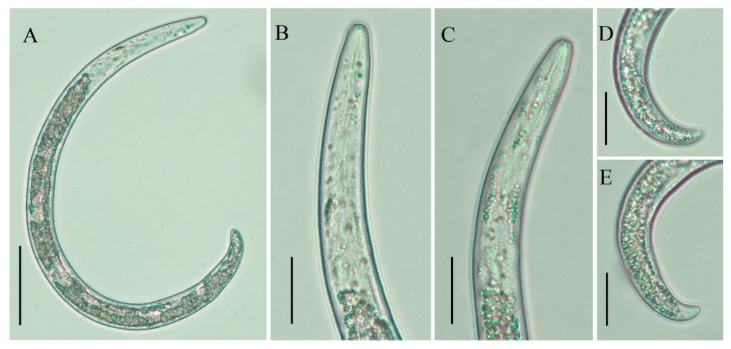
Light photomicrographs of *Paratylenchus enigmaticus* sp. nov. juvenile. (**A**) Entire body; (**B**,**C**) pharyngeal regions; (**D**,**E**) tails. Scale bars: (**A**) 50 μm; (**B**–**E**) 20 μm.

(Figure 6, Figure 7 and Figure 8; Table 3).

http://zoobank.org/urn:lsid:zoobank.org:act:39C84EDC-15ED-491E-9373-8876D34C35ED.

Body slender, ventrally arcuate to form an open, C-shaped body habitus when heat relaxed; cuticle finely annulated; lateral field equidistant with four distinct lines, outer lines are more prominent than the inner ones; lip region conoid rounded, with anterior end flattened, continuous with the rest of the body; labial framework sclerotization weak; pharyngeal region typical paratylenchoid type; stylet rigid, straight; stylet knobs rounded; dorsal pharyngeal gland opening 4.0–6.0 μm behind stylet knobs; median pharyngeal bulb slender elongate, bearing distinct large valves; isthmus short slender, surrounded by nerve ring; basal bulb pyriform, pharyngeal-intestinal valve rounded; excretory pore situated at the level or anterior to pharyngeal basal bulb; hemizonid 1–2 annuli long situated immediately posterior to excretory pore; body slightly narrower posterior to vulva; ovary outstretched, well developed; spermatheca and crustaformeria well developed; spermatheca rounded; vulva a transverse slit occupying half of the corresponding body width; vulval lips prominent, the anterior lip is protruding further than the posterior lip; vulval flaps present, but not prominent in fresh specimens; a small rudimentary post uterine branch present along the ventral body wall; anus indistinct; the tail slender, conoid, finely annulated, and gradually tapers to form a rounded terminus.

#### 2.3.1. Juvenile

Only one form was detected. This stage of individuals was similar in morphology to the adult females. However, they were characterized by the presence of weak stylet; underdeveloped pharynx components; underdeveloped genital primordium; indistinct anus; and posterior body with a rounded terminus.

#### 2.3.2. Diagnosis and Relationship

The new species is characterized by the presence of 4 lateral lines, advulval flaps, and a moderate stylet length of 28.8 (27.3–30.8) µm. The lip region is conoid rounded, with the anterior end flattened, continuous with the rest of the body. The excretory pore is situated at the level or anterior to the pharyngeal basal bulb. The spermatheca is rounded, and a small rudimentary post uterine branch is present. The tail conoid gradually tapers to form a rounded terminus.

Morphologically, the new species is close to *P. dianthus*, *P. neoprojectus*, *P. nanus* Cobb, [35] and *P. projectus*. The new species can be differentiated from *P. dianthus* by lip region morphology (conoid rounded vs. truncate), presence of small post uterine sac (vs. absent), tail terminus morphology (broadly rounded vs. finely rounded, rarely clavate, or sometimes digitate), and higher c’ value (3.5 (3.0–4.5) vs. 2.5). From *P. neoprojectus*, the new species can be differentiated by lip region morphology (conoid rounded vs. rounded), tail terminus morphology (broadly rounded vs. conically rounded), and position of excretory pore (at the level or anterior to pharyngeal bulb vs. at the level or middle of pharyngeal bulb). From *P. nanus* it differs by lip region morphology (conoid rounded vs. rounded), tail terminus morphology (broadly rounded vs. subacute to rounded, slightly indented), and shorter stylet length (28.8 (27.3–30.8) µm vs. 32–34 µm). From *P. projectus*, the new species differs by lip region morphology (conoid rounded vs. offset, conoid truncate, or trapezoid), presence of small post uterine sac (vs. absent), tail terminus morphology (broadly rounded vs. rounded dorsally sinuate), shorter stylet length (28.8 (27.3–30.8) µm vs. 25–37 µm), and higher c’ value (3.5 (3.0–4.5) vs. 2.7).

#### 2.3.3. Remarks

The species was first found (but not described) in the glasshouse-grown lettuce from Belgium. The species causes damage to the root system, but this was not related to significant yield reduction in lettuce heads [34]. In the present study, same species was found in the potato growing region of southern Alberta. In the Belgian population, the authors noted the presence of a large proportion of pre-adults 51–96% and stated this might be due to soil disturbance [34]. The Canadian population also exhibits the same feature; the juveniles were observed in higher numbers than females. Morphological, molecular, and morphometrical comparisons indicate that the Canadian and the Belgian populations are conspecific, and in this study are described as *P. enigmaticus* sp. nov.

#### 2.3.4. Type Habitat and Locality

*Paratylenchus enigmaticus* sp. nov. was found in a potato field (latitude 49°42′34.3″ N; longitude—112°3′54.1″ W); the municipal district of Taber, Alberta, Canada.

#### 2.3.5. Etymology

The species name, *enigmaticus*, refers to the species identity remaining unresolved for several months.

#### 2.3.6. Type Material

Holotype female, 9 paratypes females, and 2 juveniles (7 slides, numbers UL-DY1-01 to UL-DY1-07) and additional 5 slides containing females were deposited in the Nematode Collection of the University of Lethbridge, Alberta, Canada. Two females and three juveniles were deposited in the Nematode Collection of the Institute for Sustainable Agriculture, CSIC, Córdoba, Spain.

### 2.4. Molecular Characterization and Phylogenetic Analysis of Paratylenchus Populations from Canada and Spain

The amplification of the D2–D3 expansion domains of the 28S rRNA, ITS region, and 18S rRNA genes of *Paratylenchus* populations yielded single fragments of ~1000 bp, 800 bp, and 800 bp, respectively. Ten new sequences from the D2–D3 expansion domains of the 28S rRNA gene, 11 from ITS, and two new sequences from the 18S rRNA gene were obtained in this study.

The D2–D3 expansion domains of the 28S rRNA sequences of *P. enigmaticus* sp. nov. (MW282760–MW282761) and *Paratylenchus* sp. T1–T5 (MN535542–MN535545) from Belgium showed no intraspecific variability (100% similarity) from each other. The sequence identities of *P. enigmaticus* sp. nov. with *Paratylenchus* sp. T1–T5 from Belgium, *P. tenuicaudatus* Wu [36] (KU291239, from Iran), and *P. tateae* (MW282754–MW282759) were 99% (1 bp difference and 0 indels), 95% (38 bp difference and 1 indel), and 99% (3–4 different nucleotides and 0 indels), respectively. Similarly, the D2–D3 sequences of *P. tateae* from Canada and Spain showed low intraspecific variability (99% similarity). The sequence identities of *P. tateae* with *P. sheri* Raski [37] (MN088374, from Iran), and *P. similis* Khan, Prasad, Mathur [38] (MN088375, from Iran) were 99% (differed in 5 nucleotides and 0 indels) and 98% (differed in 16 bp and 0 indels). *Paratylenchus neoprojectus* (MW282762–MW282763) sequences obtained in this study differs in 0–7 nucleotides and 0 indels (99–100% similarity) from sequences of *P. neoprojectus* (=*P. nanus* type B) from USA (KF242201, MH790252, MH6722687, MH237651), South Korea (KY468900, KY468899, KF242199, KY468901) and South Africa (KF242200, KF242198). Finally, Canadian *P. neoprojectus* sequence differs in 10 nucleotides and 0 indels (98% similarity) from a short 542 bp sequence of *P. coronatus* Colbran [39] (MK506808) from Iran.

The ITS sequences of Canadian and Spanish populations of *P. tateae* MW282766–MW282771) showed lower intraspecific variability at 99% similarity with 3 different nucleotides and 1–2 indels. The ITS sequences of *P. neoprojectus* (MW282775–MW282776) and *P. enigmaticus* sp. nov. showed low intraspecific variability with 4 and 1–11 different nucleotides, respectively, and 0–3 indels (98–99% similarity). The ITS sequences of *P. enigmaticus* sp. nov. (MW282772–MW282774) and *Paratylenchus* sp. T1–T5 from Belgium (MN535542–MN535545) are very similar, with 97% similarity (16–17 nucleotides difference, 4 indels), whereas the other close species, i.e., *P. hamatus* (KF242253, KF242246), *P. tenuicaudatus* (KF24226, KF242261), and *Paratylenchus* sp. SAS (KF242243) from the USA showed 90–91% (60–71 nucleotides difference, 13–18 indels) similarity with *P. enigmaticus* sp. nov. The *P. neoprojectus* sequence of the Canadian population differs in 4–25 nucleotides and 0–7 indels (97–99% similarity) from sequences of *P. neoprojectus* (=*P. nanus* type B) from USA (MH236098), South Korea (MN710514, MN710515, KY468905, KY468904), and South Africa (KF242264, KF242263). The molecular information in the NCBI database regarding the 18S rRNA gene of pin nematode species is insufficient to calculate the sequence identities for this marker because few sequences have been deposited and there are not many molecular differences between species.

**Figure 9:**
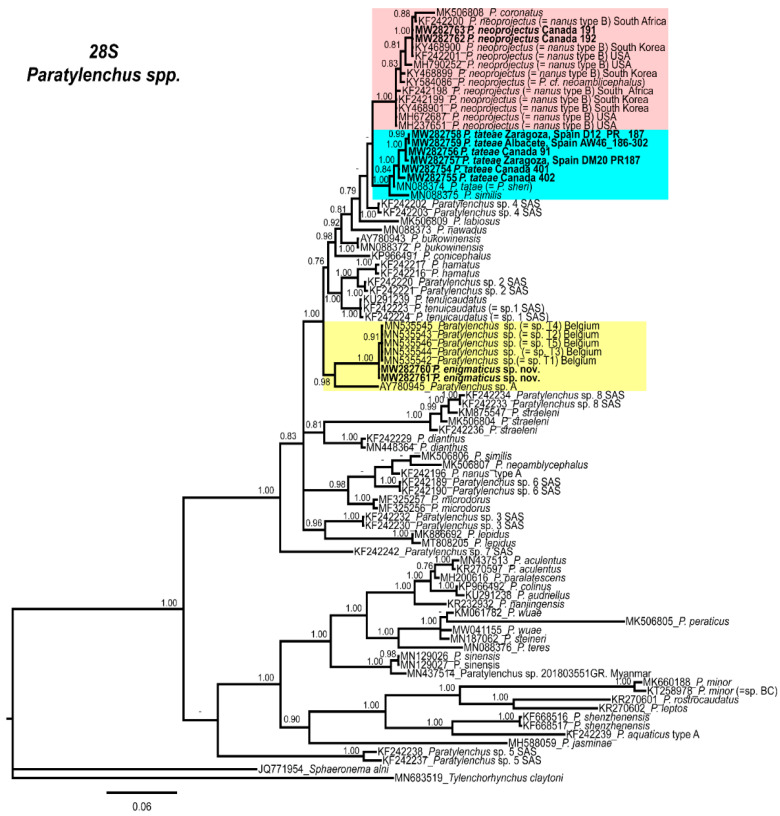
Phylogenetic relationships within the genus *Paratylenchus*. Bayesian 50% majority rule consensus tree as inferred from the D2–D3 expansion domains of the 28S rRNA sequence alignment under the general, time-reversible model of sequence evolution with correction for invariable sites and a gamma-shaped distribution (GTR + I+ G). Posterior probabilities of more than 0.70 are given for appropriate clades. Newly obtained sequences in this study are shown in bold. The scale bar indicates expected changes per site.

**Figure 10:**
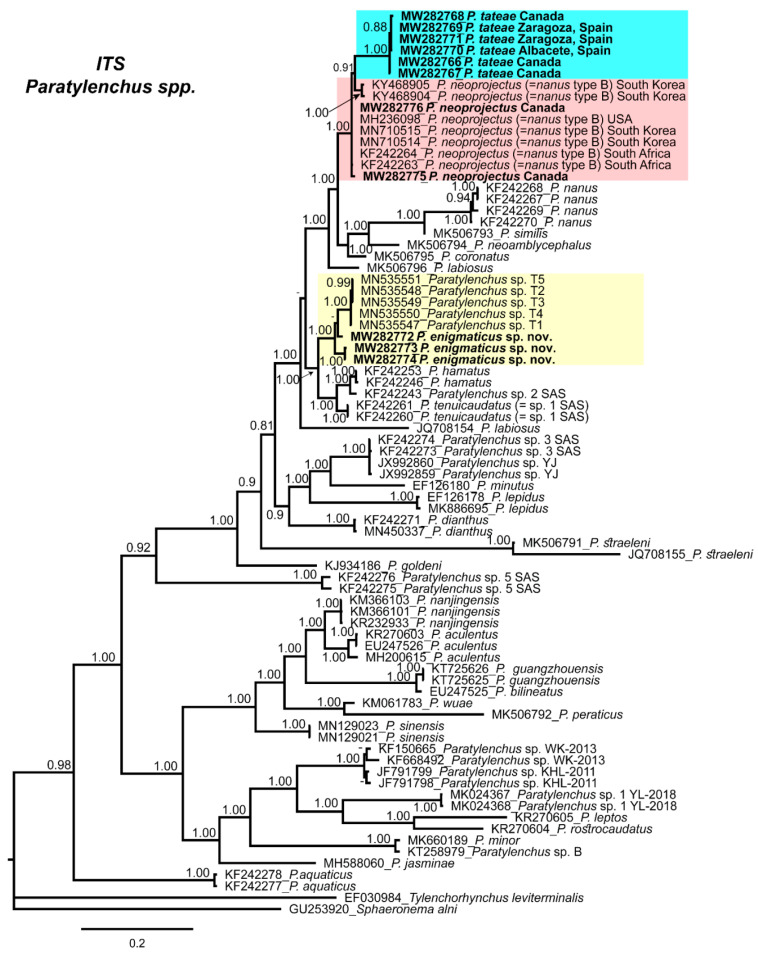
Phylogenetic relationships within the genus *Paratylenchus*. Bayesian 50% majority rule consensus tree as inferred from ITS rRNA sequence alignment under the general, time-reversible model of sequence evolution with correction for invariable sites and a gamma-shaped distribution (GTR + I+ G). Posterior probabilities greater than 0.70 are given for the corresponding clades. Newly obtained sequences in this study are shown in bold. The scale bar indicates expected changes per site.

**Figure 11:**
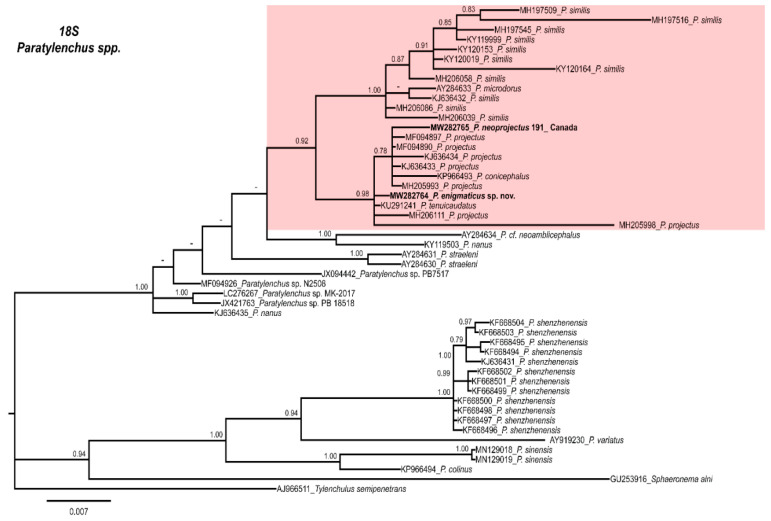
Phylogenetic relationships within the genus *Paratylenchus*. Bayesian 50% majority rule consensus tree as inferred from the partial 18S rRNA sequence alignment under the general, time-reversible model of sequence evolution with correction for invariable sites and a gamma-shaped distribution (GTR + I+ G). Posterior probabilities greater than 0.70 are given for appropriate clades. Newly obtained sequences in this study are shown in bold. The scale bar indicates expected changes per site.

Phylogenetic relationships among *Paratylenchus* species inferred from analyses of the D2–D3 expansion domains of 28S rRNA, ITS region, and partial 18S rRNA sequences using BI are shown in Figure 9, Figure 10 and Figure 11, respectively. The phylogenetic trees generated from the three nuclear markers, included 89, 81, and 50 sequences, with 680, 875, and 1610 nucleotides, respectively.

The D2–D3 expansion domains of the 28S rRNA phylogenetic tree of *Paratylenchus* spp. showed two main clades, one highly supported (PP = 1.00), including the three species described in this study, and another weakly supported (PP = 0.51), including several *Paratylenchus* spp.; most of them with a longer stylet (>40 µm; Figure 9). The *P. enigmaticus* sp. nov. clustered together in a highly supported subclade (PP = 1.00) with sequences of *Paratylenchus* sp. T1–T5 from Belgium, and was well separated (PP = 0.98) from *Paratylenchus* sp. A (AY780945) from California, USA (Figure 9). Moreover, *P. neoprojectus* clustered together in a highly supported subclade (PP = 1.00) with sequences of *P. neoprojectus* (=*P. nanus* type B) and *P. coronatus* (MK506808). It is also noted that the sequence of *P. sheri* (MN088374) provided by Mirbabaei et al. [40] grouped with the Canadian and Spanish populations of *P. tateae*. The molecular identities suggest that this sequence belongs to *P. tateae* instead of *P. sheri*. The morphological and molecular details associated with the *P. sheri* sequence suggest a possible error in the sequencing. It is therefore recommended to use the same specimen for morphological and molecular studies. Consequently, we consider MN088374 as *P. tateae* in our study.

The 50% majority rule consensus ITS BI tree also shows 2 clades, one representing short stylet species, including the three species described in this study, and the second containing mostly long stylet species (Figure 10). Likewise, the D2–D3 expansion domains of the 28S rRNA tree, *P. enigmaticus* sp. nov. grouped with *Paratylenchus* sp. T1–T5 from Belgium (PP = 1.00), and shares a clade with *P. hamatus*, *P. tenuicaudatus*, and *Paratylenchus* sp. SAS. Canadian and Spanish populations of *P. tateae* grouped with several populations of *P. neoprojectus* (PP = 0.91).

Finally, the phylogenetic relationships of *Paratylenchus* species inferred from analysis of partial 18S rRNA gene sequences shows two clades that are well defined (Figure 11), but several subclades that do not resolve well in the clade include *P. enigmaticus* sp. nov. (MW282764) and *P. neoprojectus* (MW282765).

## 3. Discussion

*Paratylenchus* is a large genus that comprises short and long stylet species [23]. The majority of short stylet species are considered pathogenic and cause significant damage to their host plants [22]. So far, six short stylet species from Canada have been reported, namely *P. brevihastus*, *P. labiosus*, *P. neoprojectus*, *P. projectus*, *P. tateae*, and *P. tenuicaudatus*. All of these are Canadian native species except *P. projectus,* which is a cosmopolitan species known to have a global distribution [23].

Morphological identification of *Paratylenchus* species is difficult because of their variable characters and overlapping morphometrical values. Stylet length, number of lateral lines, and presence/absence of vulva flaps are considered to be robust characters for species differentiation; however, body length, tail length and shape, position of excretory pore, and ratios of c, c’ were concluded to be unreliable for species separation [25,41,42]. As the majority of *Paratylenchus* species presents a limited selection of differences in morphology, several nematologists have attempted to synonymize morphologically similar species. For example, Brzeski [43] synonymized *P. tateae*, *P. labiosus*, and *P. italiensis* with *P. similis,* because of their similar morphology and overlapped morphometrical values. Ghaderi et al. [25] accepted the synonymization of *P. similis* and *P. tateae*; however, with the availability of molecular data, the same authors [23] rejected the change and referred to both species as valid taxa, and also commented that several populations of *P. similis* may indeed be *P. tateae*. Bahmani et al. [44] also presented a detailed argument on the validity of *P. labiosus,* which was supported by molecular data in Mirbabaei et al. [40].

The possible presence of species complexes in pin nematodes was highlighted by Van den Berg et al. [28] and Mirbabaei et al. [40]. We are in agreement with the authors that similar appearances and overlapping morphometrical characters may present difficulties in ascertaining species status. Nevertheless, such morphological complexes can be resolved using molecular data. Several taxonomic issues have been successfully addressed with molecular studies, such as the validity and differentiation of *Radopholoides* from *Hoplotylus* and *Radopholus* [45], the transfer of *Tylaphelenchus jiaae* to the genus *Pseudaphelenchus* as *P. jiaae* [46], the revision and species synonymization in *Laimaphelenchus* [47], the species delimitation in members of Criconematoidea [48,49,50,51], and the resolution of the cryptic diversity and species complexes in Longidoridae [52,53,54].

Our phylogenetic analysis of D2–D3 expansion domains of the 28S rRNA also indicates that the status of *P. nanus* type B [28] and *P. sheri* [40] need detailed revision. By comparing all the available molecular and morphometric data from both species, it is evident that *P. nanus* type B is a population of *P. neoprojectus* and *P. sheri* is a population of *P. tateae*. Additionally, our *P. enigmaticus* sp. nov. appears conspecific with the Belgian population (T1–T5). It is notable that molecular data not only resolve the taxonomic issues, but also aids in eliminating the propagation of redundant data.

In the literature, several studies have outlined a wide host range [55,56,57] and survival abilities of pin nematodes [58,59]. Biologically, the final juvenile stage of certain species of pin nematode constitutes the highest portion of the total population. Rhoades and Linford [58] and Wood [15] refer to this stage as a resistant non-feeding stage which is more capable of withstanding desiccation and sudden freezing than the younger and adult stages.

The Canadian and Belgian populations of *P. enigmaticus* sp. nov. have a higher proportion of juveniles than adults, whereas *P. tateae* and *P. neoprojectus* have higher quantities of females than juveniles. It appears that *P. enigmaticus* sp. nov. has a resistant stage; however, the presence of such a stage needs confirmation through further study.

There are limited data regarding the prevalence of pin nematodes in the potato growing areas of southern Alberta and other parts of Canada. Thus far, *P. labiosus* and *P. projectus* are the only species detected in the potato growing areas of Prince Edward Island and New Brunswick [13,32,60,61]. In the present study, we identified *P. neoprojectus*, *P. tateae*, and *P. enigmaticus* sp. nov. in southern Alberta, along with *P. tateae* populations from Spain, using an integrative taxonomical approach. Our study also underscores the importance of using molecular data for accurate species identification and clarifying the status of *P. nanus* type B and *P. sheri*.

Lower densities of identified species in the samples suggest that these are mild parasitic species and, as of yet, do not behave as potential pests. However, pin nematodes have a reputation of building high population densities in short periods, and, under favorable circumstances, can be a threat to their hosts [22,34]. Indeed, a higher incidence of root lesion nematodes (*Pratylenchus* spp.) in southern Alberta has been reported by Forge et al. [4]. Having that in mind, the densities of pin nematodes are worth monitoring as some species can penetrate roots through existing entry points and may aggravate the plant damage. Therefore, further studies are required to assess species-specific yield losses and thresholds.

## 4. Materials and Methods

### 4.1. Isolation and Morphological/Morphometrical Studies

Nematodes were extracted from soil samples using the modified Cobb sieving and flotation-centrifugation method [62]. For preliminary examinations, fresh nematodes were transferred to the drop of distilled water, heat relaxed at 60 °C for 30–45 s, and observed under the Zeiss Axioskope 40 microscope. Permanent mounts were prepared as described in Seinhorst [63] and De Grisse [64]. Light micrographs of the mounted specimens were acquired using a Zeiss Axioskope 40 microscope equipped with a Zeiss Axiocam 208 camera (Carl Zeiss Microscopy, Jena, Germany). Standard morphometrical characters were selected based on previously published studies [25,28,57,65]. Measurements were made using ZEN blue 3.1 imaging software (Carl Zeiss Microscopy).

### 4.2. DNA Extraction, PCR and Sequencing

Nematode DNA was prepared according to Maria et al. [65]. Three sets of DNA primers (Integrated DNA Technologies, Coralville, IA, USA) were used in the PCR analyses to amplify the nucleotide sequences of the partial 18S, D2–D3 expansion domains of the 28S rRNA and ITS of ribosomal genes, including 5.8S rRNA and both ITS regions (ITS1 and ITS2) (rRNA). The partial 18S rRNA region was amplified with 1813F and 2646R primers [66]. The D2–D3 expansion domains of the 28S rRNA regions were amplified using 28–81F and 28–1006rev primers [67], and the ITS region was amplified using F194 [68] and AB28 primers [69]. The ribosomal gene cluster (whole rDNA cistron) is a multicopy, tandem repeated array in the genome. Each repeat is transcribed as a single rRNA precursor and cleaved, leading to the mature small subunit rRNA (SSU), the mature 5.8S rRNA, and the mature large subunit rRNA (LSU). The SSU is separated from the 5.8S rRNA by the first internal transcribed spacer (ITS1), and the second internal transcribed spacer (ITS2) is located between the 5.8S rRNA and the LSU [70]. A nice scheme of these repeats and the position of many of the primers used by nematologists could be found in Carta and Li [71]. The PCR conditions were as described in Holterman et al. [66,67] and in Ferris et al., [68]. Amplified PCR products were resolved by electrophoresis in 1% agarose gels and visualized by staining with GelRed (Biotium, Fremont, CA, USA). Amplified DNA fragments were purified using an E.Z.N.A Gel Extraction kit (Omega Biotek, Norcross, GA, USA), following manufacturer’s instructions, ligated into the pJET1.2 vector (Thermo Fisher Scientific, Mississauga, ON, Canada), and introduced into *Escherichia coli* DH5α competent cells (Thermo Fisher Scientific). The presence of the PCR-derived inserts in the plasmids from transformed *E. coli* cells was confirmed by PCR. Plasmid DNA was isolated and purified using E.Z.N.A Plasmid DNA minikit I (Omega Biotek), according to the manufacturer’s instructions, and sent to Genewiz, Inc for DNA sequencing (South Plainfield, NJ, USA). DNA sequences were aligned using the Bioedit sequence alignment tool and compared for similarities with all known nematode species sequences in the GenBank database.

### 4.3. Phylogenetic Analyses

Sequenced genetic markers from the nematodes examined in the present study (after discarding primer sequences and ambiguously aligned regions) and several pin nematode sequences obtained from GenBank were used in the phylogenetic reconstruction. Outgroup taxa for each dataset were selected based on previously published studies [57]. Multiple sequence alignments of the newly obtained and published sequences were made using the FFT-NS-2 algorithm of MAFFT V.7.450 [72]. Sequence alignments were visualized with BioEdit [73] and manually edited using Gblocks ver. 0.91b [74] in the Castresana Laboratory server (http://molevol.cmima.csic.es/castresana/Gblocks_server.html) using options for a less stringent selection (minimum number of sequences for a conserved or a flanking position: 50% of the number of sequences +1; maximum number of contiguous non-conserved positions: 8; minimum length of a block: 5; allowed gap positions: With half).

Phylogenetic analyses of the sequence datasets were conducted based on Bayesian inference (BI) using MRBAYES 3.2.7a [75]. The best-fit model of DNA evolution was calculated with the Akaike information (AIC) of JMODELTEST V.2.1.7 [76]. The best-fit model, base frequency, proportion of invariable sites, substitution rates and gamma distribution shape parameters in the AIC were used for phylogenetic analyses. BI analyses were performed under a general time reversible model, with a proportion of invariable sites and a rate of variation across sites (GTR + I + G) for the partial 18S rRNA, D2–D3 expansion domains of the 28S rRNA, and ITS region sequences. These BI analyses were run separately per dataset with four chains for 2 × 10^6^ generations. The Markov chains were sampled at intervals of 100 generations. Two runs were conducted for each analysis. After discarding burn-in samples of 20% and evaluating convergence, the remaining samples were retained for more in-depth analyses. The topologies were used to generate a 50% majority-rule consensus tree. Posterior probabilities (PP) are given on appropriate clades. Trees from all analyses were edited using FigTree software V.1.4.4 (http://tree.bio.ed.ac.uk/software/figtree/).

## Figures and Tables

**Table 1:** Morphometrics of *Paratylenchus neoprojectus* females and juveniles. All measurements are in µm and presented as mean ± standard deviation (range).

	Present Study		Wu & Hawn [26]	* Van den Berg et al. [28]	
Characters	Females	Juveniles	Females	Females	Juveniles
n	11	4	76	17	4
Body length	383.5 ± 36.7 (330.0–434.0)	342.0 ± 19.6 (322.0–365.0)	327–405	359 (300–415)	339.5 (299–390)
a	24.0 ± 1.7 (21.0–26.0)	22.3 ± 1.9 (20.5–24.3)	18–26	22.1 (19.5–24.6)	20.4 (17.7–22.9)
b	3.8 ± 0.3 (3.3–4.3)	3.9 ± 0.3 (3.5–4.1)	3.8–4.6	3.9 (3.5–4.4)	4.1 (3.7–4.7)
c	14.6 ± 1.8 (12.1–18.5)	12.8 ± 1.6 (11.0–15.0)	14–16	15.3 (14–18.5)	13.8 (12.3–18.9)
c’	2.7 ± 0.2 (2.3–3.0)	2.3 ± 0.3 (1.9–2.6)	-	2.4 (2.0–2.8)	2.2 (1.7–2.5)
V	84.4 ± 1.3 (82.0–85.8)	-	82–85.7	84 (82.5–85)	-
Stylet percentage	7.0 ± 0.8 (5.8–8.3)	-	-	8 (6.8–9.3)	-
Lip height	3.3 ± 0.4 (3.0–4.0)	-	-	3.5 (3–4)	-
Lip width	6.4 ± 0.4 (6.0–7.0)	-	-	7 (6.5–7.5)	-
Stylet length	25.3 ± 1.3 (25.0–29)	13.3 ± 1.0 (12.0–14.0)	28–31	28.5 (26–31)	10 (3.5–14.5)
Median bulb length	23.4 ± 1.6 (21.0–25.0)	-	-	-	-
Median bulb width	9.3 ± 0.8 (8.0–11.0)	-	-	-	-
Anterior end to excretory pore	79.1 ± 4.8 (70.0–85.0)	75.0 ± 5.2 (70.0–80.0)	-	77.5 (71–85)	71 (65–78.5)
Pharynx length	99.0 ± 4.2 (92.0–106.0)	89.0 ± 6.2 (80.0–93.0)	82–94	92 (85–110)	83.5 (72.5–94.5)
Maximum body width	16.0 ± 1.4 (13.5–18.0)	15.4 ± 0.4 (15.0–15.8)	-	16 (13–20)	-
Vulva body width	13.6 ± 1.3 (12.0–15.0)	-	-	-	-
Anal body width	9.7 ± 0.9 (8.0–11.2)	11.7 ± 0.7 (10.7–12.4)	-	-	-
Distance from vulva to anus	33.5 ± 5.8 (28.0–44.0)	-	29–44	33.5 (26–44)	-
Distance from vulva to tail terminus	60.0 ± 7.3 (50.0–72.0)	-	-	-	-
Tail length	26.0 ± 2.9 (22.0–30.0)	27.0 ± 3.5 (22.0–30.0)	23–27	23.5 (17.5–29.5)	23 (20.5–29.5)

* Van den Berg et al. [28] represent the measurements of *P. nanus* type B. In this study, we refer this population as *P. neoprojectus*.

**Table 2:** Morphometrics of Canadian and Spanish populations of *Paratylenchus tateae*. All measurements are in µm and presented as mean ± standard deviation (range).

	Canadian Populations			Spanish Populations		Wu & Townshend [27]
Characters	Females		Juveniles	Females		Females
Populations	091	041	091	Ariza, Zaragoza	Alpera, Albacete	type population
n	18	18	6	20	8	43
Body length	333.6 ± 33.7 (269.0–380.0)	349.5 ± 25.4 (314.0–388.0)	315.5 ± 26.4 (267.0–342.0)	346.2 ± 25.8 (310.0–425.0)	334.4 ± 14.3 (310.0–353.0)	315–401
a	23.4 ± 1.4 (21.4–26.2)	23.9 ± 1.9 (20.4–27.0)	22.0 ± 1.9 (18.8–24.0)	21.8 ± 1.6 (17.4–24.3)	21.7 ± 1.7 (19.1–23.5)	19–26
b	3.6 ± 0.3 (3.2–4.0)	3.9 ± 0.3 (3.3–4.7)	3.9 ± 0.2 (3.5–4.1)	3.7 ± 0.2 (3.3–4.2)	3.6 ± 0.2 (3.3–4.1)	3.8–5.9
c	11.9 ± 1.1 (10.0–13.8)	13.5 ± 1.4 (11.6–16.9)	15.4 ± 1.9 (13.5–18.9)	13.9 ± 1.9 (10.5–17.7)	13.2 ± 1.8 (11.3–15.3)	11.7–15.8
c’	3.5 ± 0.4 (3.0–4.5)	3.3 ± 0.3 (2.8–3.9)	2.6 ± 0.1 (2.4–2.8)	2.9 ± 0.4 (2.5–3.8)	2.9 ± 0.2 (2.6–3.1)	-
V	82.3 ± 1.2 (80.0–84.3)	82.9 ± 0.9 (80.8–84.1)	-	82.9 ± 1.4 (80.2–85.6)	82.6 ± 1.4 (81.3–85.0)	80.5–84.7
Lip height	2.6 ± 0.2 (2.0–3.0)	2.8 ± 0.3 (2.0–3.0)	-	-	-	-
Lip width	5.5 ± 0.2 (5.0–6.0)	5.6 ± 0.4 (5.0–6.0)	-	5.2 ± 0.4 (4.5–6.0)	5.2 ± 0.4 (4.5–6.0)	-
Stylet length	17.3 ± 0.9 (15.0–19.0)	16.5 ± 0.9 (14.5–18.0)	12.0 ± 1.1 (10.0–13.0)	15.5 ± 0.4 (14.5–16.0)	15.4 ± 0.4 (15.0–16.0)	15–16.8
Median bulb length	21.9 ± 1.5 (19.4–24.2)	20.6 ± 2.3 (16.0–24.0)	-	18.2 ± 1.7 (15.5–22.0)	17.4 ± 1.2 (16.0–19.0)	-
Median bulb width	8.1 ± 0.6 (7.2–9.0)	8.2 ± 0.8 (7.2–10.0)	-	8.9 ± 0.6 (8.0–10.0)	8.6 ± 0.5 (8.0–9.5)	-
Anterior end to excretory pore	73.9 ± 3.8 (64.0–81.0)	73.4 ± 5.3 (63.0–84.0)	66.8 ± 4.8 (60.0–71.0)	78.2 ± 6.0 (70.5–93.0)	77.4 ± 3.8 (72.5–84.0)	68–81
Pharynx length	91.7 ± 3.4 (83.0–98.0)	90.2 ± 4.9 (82.0–98.0)	80.3 ± 2.9 (76.0–83.0)	93.1 ± 5.0 (85.5–103.0)	92.1 ± 5.6 (85.5–102.0)	77–89
Maximum body width	14.2 ± 1.2 (12.0–16.0)	14.6 ± 0.8 (13.0–16.0)	14.3 ± 0.6 (13.0–15.0)	16.0 ± 1.9 (14.5–21.5)	15.5 ± 1.3 (14.5–18.5)	-
Anal body width	8.1 ± 0.8 (6.0–9.0)	7.9 ± 0.6 (7.0–9.0)	7.9 ± 0.2 (7.5–8.0)	8.7 ± 0.4 (8.0–9.5)	8.9 ± 0.9 (8.0–11.0)	-
Distance from vulva to anus	30.6 ± 3.6 (26.0–39.0)	33.5 ± 4.2 (27.0–43.0)	-	-	-	28–41
Distance from vulva to tail terminus	58.8 ± 5.6 (52.0–70.6)	59.6 ± 4.8 (51.0–67.0)	-	-	-	-
Tail length	28.2 ± 3.0 (24.0–35.0)	26.1 ± 2.5 (21.0–30.0)	20.7 ± 1.7 (18.0–23.0)	25.3 ± 3.3 (21.5–32.5)	25.6 ± 2.9 (22.5–30.0)	22–33

**Table 3:** Morphometrics of Canadian and Belgian populations of *Paratylenchus enigmaticus* sp. nov. All measurements are in µm and presented as mean ± standard deviation (range).

	Canadian Population			* Belgian Population Claerbout et al. [34]				
	Holotype	Paratype						
Characters	Female	Females	Juveniles	T1	T2	T3	T4	T5
n		11	5	10	10	10	10	10
Body length	372	382.7 ± 30.9 (343.0–431.0)	344.3 ± 9.5 (331.0–357.0)	365 ± 40 (308–465)	335 ± 20 (302–360)	365 ± 39 (313–422)	358 ± 43 (300–411)	328 ± 31 (293–368)
a	24.6	25.7 ± 2.1 (21.7–28.7)	23.8 ± 0.4 (23.1–24.4)	24.2 ± 3.8 (14.9–27.6)	24.3 ± 3.4 (19.3–27.2)	26.7 ± 2.3 (22–29)	23.7 ± 2.6 (18.5–27.5)	23.2 ± 3.3 (18.1–28.1)
b	3.9	4.1 ± 0.3 (3.7–4.7)	4.2 ± 0.2 (3.9–4.4)	3.7 ± 0.7 (2.7–4.6)	-	3.4 ± 0.7 (2.5–4.9)	3.2 ± 0.5 (2.8–4.2)	-
c	15.7	15.4 ± 1.3 (12.9–17.5)	14.9 ± 0.5 (14.4–15.7)	15.0 ± 1.5 (12.3–17.2)	14.9 ± 1.5 (13.2–17)	14.9 ± 1.9 (12.7–17.8)	14.8 ± 2.3 (13.7–19.8)	13.0 ± 1.5 (10.1–15.7)
c′	2.5	2.6 ± 0.3 (2.3–3.1)	2.3 ± 0.3 (1.9–2.6)	-	-	-	-	-
V	84.1	85 ± 0.9 (83.0–86.3)	-	83.2 ± 2.1 (80.4–87.8)	83.2 ± 2.1 (80–87)	83.0 ± 1.5 (80–84)	83.5 ± 0.9 (82.8–84.9)	83.1 ± 2.1 (80.1–88)
Lip height	3.1	3.0 ± 0.3 (2.6–3.6)	-	-	-	-	-	-
Lip width	7.5	7.1 ± 0.4 (6.5–7.7)	-	-	-	-	-	-
Stylet length	28.9	28.8 ± 1.1 (27.3–30.8)	12.5 ± 0.9 (11.2–13.5)	27.3 ± 1.3 (23.5–28.4)	25.5 ± 1.6 (22.3–26.5)	26.6 ± 1.5 (25.2–30.5)	26.8 ± 1.3 (24.6–27.9)	27.0 ± 1.5 (24.6–28.6)
Stylet percentage	7.7	7.6 ± 0.5 (6.8–8.2)	-	7.5 ± 0.9 (6.0–8.8)	7.6 ± 0.7 (7.2–8.8)	7.3 ± 0.7 (6.2–7.9)	7.6 ± 0.8 (6.6–8.4)	8.3 ± 0.5 (7.3–8.9)
Median bulb length	21.2	20.4 ± 1.0 (18.5–21.3)	-	-	-	-	-	-
Median bulb width	9.8	9.6 ± 1.1 (8.0–11.4)	-	-	-	-	-	-
Anterior end to excretory pore	79	76.0 ± 4.2 (70.0–82.0)	65.2 ± 2.8 (63.0–70.0)	-	-	-	-	-
Pharynx length	95	93.8 ± 5.2 (83.0–100.0)	81.6 ± 4.3 (76.0–88.0)	100.7 ± 19.7 (75.2–137.7)	88.0 ± 23.3 (42.9–105.8)	109.9 ± 16.9 (83.3–123.5)	114.7 ± 18.4 (84.6–125.7)	120.4 ± 14.6 (95.0–144.0)
Maximum body width	15.1	15.0 ± 1.2 (12.6–16.4)	14.4 ± 0.3 (14.2–14.9)	-	-	-	-	-
Vulva body width	12.7	13.1 ± 1.0 (11.4–14.7)	-	-	-	-	-	-
Anal body width	9.5	9.7 ± 0.9 (7.7–10.6)	10.2 ± 1.2 (8.8–11.7)	-	-	-	-	-
Distance from vulva to anus	36	33.3 ± 4.0 (26.0–37.0)	-	-	-	-	-	-
Distance from vulva to tail terminus	59.6	59.9 ± 3.1 (53.4–65.0)	-	-	-	-	-	-
Tail length	23.6	24.9 ± 2.1 (22.0–29.0)	23.2 ± 0.8 (22.0–24.0)	24.4 ± 3.1 (21.7–30.8)	22.6 ± 1.6 (20.3–26.2)	24.6 ± 1.8 (21.0–26.1)	24.5 ± 3.3 (21.2–23.7)	25.4 ± 2.6 (22.0–30.0)

* Belgian populations (T1–T5) represent measurement of females.

## Data Availability

The datasets generated during and/or analyzed during the current study are available from the corresponding author on reasonable request.
